# Melanopsin DNA aptamers can regulate input signals of mammalian circadian rhythms by altering the phase of the molecular clock

**DOI:** 10.3389/fnins.2024.1186677

**Published:** 2024-04-17

**Authors:** Kazuo Nakazawa, Minako Matsuo, Yo Kikuchi, Yoshihiro Nakajima, Rika Numano

**Affiliations:** ^1^Department of Applied Chemistry and Life Science, Toyohashi University of Technology, Toyohashi, Aichi, Japan; ^2^TechnoPro, Inc., Tokyo, Japan; ^3^Institute for Research on Next-Generation Semiconductor and Sensing Science, Toyohashi University of Technology, Toyohashi, Aichi, Japan; ^4^Health and Medical Research, National Institute of Advanced Industrial Science and Technology (AIST), Takamatsu, Kagawa, Japan

**Keywords:** melanopsin (OPN4), DNA aptamer, Melapt, circadian rhythm, molecular clock, phase shift, period gene

## Abstract

DNA aptamers can bind specifically to biomolecules to modify their function, potentially making them ideal oligonucleotide therapeutics. Herein, we screened for DNA aptamer of melanopsin (OPN4), a blue-light photopigment in the retina, which plays a key role using light signals to reset the phase of circadian rhythms in the central clock. Firstly, 15 DNA aptamers of melanopsin (Melapts) were identified following eight rounds of Cell-SELEX using cells expressing melanopsin on the cell membrane. Subsequent functional analysis of each Melapt was performed in a fibroblast cell line stably expressing both *Period2:ELuc* and melanopsin by determining the degree to which they reset the phase of mammalian circadian rhythms in response to blue-light stimulation. *Period2* rhythmic expression over a 24-h period was monitored in *Period2:ELuc* stable cell line fibroblasts expressing melanopsin. At subjective dawn, four Melapts were observed to advance phase by >1.5 h, while seven Melapts delayed phase by >2 h. Some Melapts caused a phase shift of approximately 2 h, even in the absence of photostimulation, presumably because Melapts can only partially affect input signaling for phase shift. Additionally, some Melaps were able to induce phase shifts in *Per1::luc* transgenic (Tg) mice, suggesting that these DNA aptamers may have the capacity to affect melanopsin *in vivo*. In summary, Melapts can successfully regulate the input signal and shifting phase (both phase advance and phase delay) of mammalian circadian rhythms *in vitro* and *in vivo*.

## Introduction

Most living organisms exposed to sunlight have evolved a ~ 24-h internal clock, known as a circadian rhythm, and synchronize the phases of this autonomous clock according to environmental clues (Ralph et al., 1990; Pittendrigh, 1993; Davidson et al., 2003; Yoo et al., 2004). Therefore, each organism resets the phase of its circadian rhythm to match the environmental light and dark cycle each morning. In mammals, circadian rhythms are influenced primarily by light, an input signal perceived through the eyes, which is capable of resetting the phase of transcriptional oscillations of clock genes (Takahashi, 1995; Menaker, 2003). The central pacemaker of mammalian circadian rhythms is located in the suprachiasmatic nucleus (SCN). The retinohypothalamic tract (RHT) immediately transmits information about blue light in the early morning to the SCN via the photoreceptor melanopsin (OPN4) in the retina and resets the phase of the circadian clock along the environmental cycle. Melanopsin mainly transfers environmental blue-light signals to the central clock early in the morning to reset the phase of the clock in the SCN (Hattar et al., 2003; Lucas et al., 2003; Foster, 2005; Pulivarthy et al., 2007; Ukai et al., 2007; Guler et al., 2008; LeGates et al., 2012).

Melanopsin is a photoreceptor protein expressed in retinal ganglion cells that absorbs blue light with a maximum absorbance of 477 nm. Although the exact function of melanopsin was unclear until melanopsin-knockout (KO) mice were generated in 2003, melanopsin was known to play an important role in resetting the phase of the mammalian circadian clock by blue light. In the first step, the melanopsin receptor is activated by binding *trans*-isomerized retinal ligands under blue light through the Gq family. The photosignal affects SCN neurons through Gq family-mediated transient receptor potential polycystin (TRPP) control of cell firing, glutamate transmitters, and pituitary adenylate cyclase-activating polypeptide (PACAP). The transmitted stimuli eventually increase calcium ion flux in the cytoplasm of cells in the SCN to activate protein kinase A (PKA) and PKC. These kinases phosphorylate cAMP response element-binding protein (CREB) and activate the connected to the cAMP response element (CRE) consensus sequence in the *Period 1* (*Per1*) promoter to transiently induce *Per1* transcription and reset the phase of circadian rhythms (King et al., 1997; Sun et al., 1997; Tei et al., 1997; Hida et al., 2000; Yamazaki et al., 2000).

The phase of the molecular circadian clock is reset by, and depends on, the timing of light stimulation and transient induction of *Per1* by the melanopsin photoreceptor. Autonomous circadian clocks in individual cells of both the SCN and peripheral clocks are constituted by transcription and translation feedback loops involving clock genes, such as *Clock, Bmal1, Per1-3,* and *Cryptochromes*, as well as their protein products (Albrecht et al., 1997; Antoch et al., 1997; King et al., 1997; Shearman et al., 1997; Takumi et al., 1998; Zylka et al., 1998; Kume et al., 1999; Bunger et al., 2000; Numano et al., 2006). The photosignal by melanopsin transiently induces transcription of photoreactive clock genes such as *Per1* and *Per2* in the SCN from 30 min to 2 h following delivery of a light pulse to the retina. Induction of transient expression of these input genes influences the transcriptional and translational feedback loop within a 24-h period as well as the phase of the autonomous clock (Ellington and Szostak, 1990; Albrecht et al., 1997; Shearman et al., 1997; Shigeyoshi et al., 1997; Giebultowicz, 2004; Yan and Silver, 2004).

DNA aptamers are short, single-stranded RNA/DNA molecules that can bind selectively to specific targets, proteins, peptides, and other molecules and can be used clinically to switch the function of target molecules. The main advantages of these aptamers are high target specificity, lack of immunogenicity, and ease of synthesis.

The function of the melanopsin protein is easily modified by the DNA aptamer because it is located on the cell membrane. DNA aptamers are powerful pharmaceutical agents because, unlike antibodies, they can be stored stably and duplicated easily in large quantities using PCR.

People with sleep–wake phase disorder and shift workers who only sleep for 1–4 h may have difficulty falling asleep and waking up immediately in the morning (Takahashi et al., 2008; Gentry et al., 2021). Thus, it would be socially and economically advantageous to improve the sleep–wake cycle indirectly by manipulating the ability of melanopsin to input signals into a central clock. Antagonists of melanopsin acquired via chemical screening of chemical libraries primarily contribute to delaying the rhythm phase (Jones et al., 2013).

In this study, we used the cell systematic evolution of ligands by exponential enrichment (Cell-SELEX) method to identify DNA aptamers (single-stranded DNA; ssDNA) that caused melanopsin (expressed on the cell membrane) to shift the phase of circadian rhythms (Tuerk and Gold, 1990; Hida et al., 2000; Yamazaki et al., 2000; Umekage and Kikuchi, 2006; Dua et al., 2011). In total, 15 types of melanopsin aptamers were analyzed to assess their ability to shift the phase of *Per2::ELuc* bioluminescent oscillations in *Per2:ELuc:*TK:Mel stable cells, in which a bioluminescent reporter follows the *Per2* promoter region controlling an enhanced green-emitting luciferase from *Pyrearinus termitilluminans*, with melanopsin overexpressed under the control of the thymidine kinase (TK) promotor (Nakajima et al., 2010). In these stable fibroblast cell lines, the signal pathway is incorporated into a fibroblast cell mimicking the signal pathway from the retina to the SCN by melanopsin.

Among these 15 DNA aptamers of melanopsin (Melapts), four Melapts induced phase advance and seven Melapts induced delay of circadian rhythms (by >1.5 h and > 2 h, respectively) in the *Per2::ELuc* cell line. Some Melapts induced phase shifts of ~2 h even in the absence of photostimulation *in vitro*. As the results from *Per1::luc* transgenic (Tg) mice were similar to the *in vitro* results from the *Per2::ELuc* cell line, melanopsin was used to induce phase Shifts *in vivo*. In summary, Melapts were able to regulate input signals and phase shifts to achieve both phase advance and phase delay of mammalian circadian rhythms *in vitro* and *in vivo*.

## Materials and methods

### Screening of DNA aptamers by the cell-SELEX method

**Figure 1:**
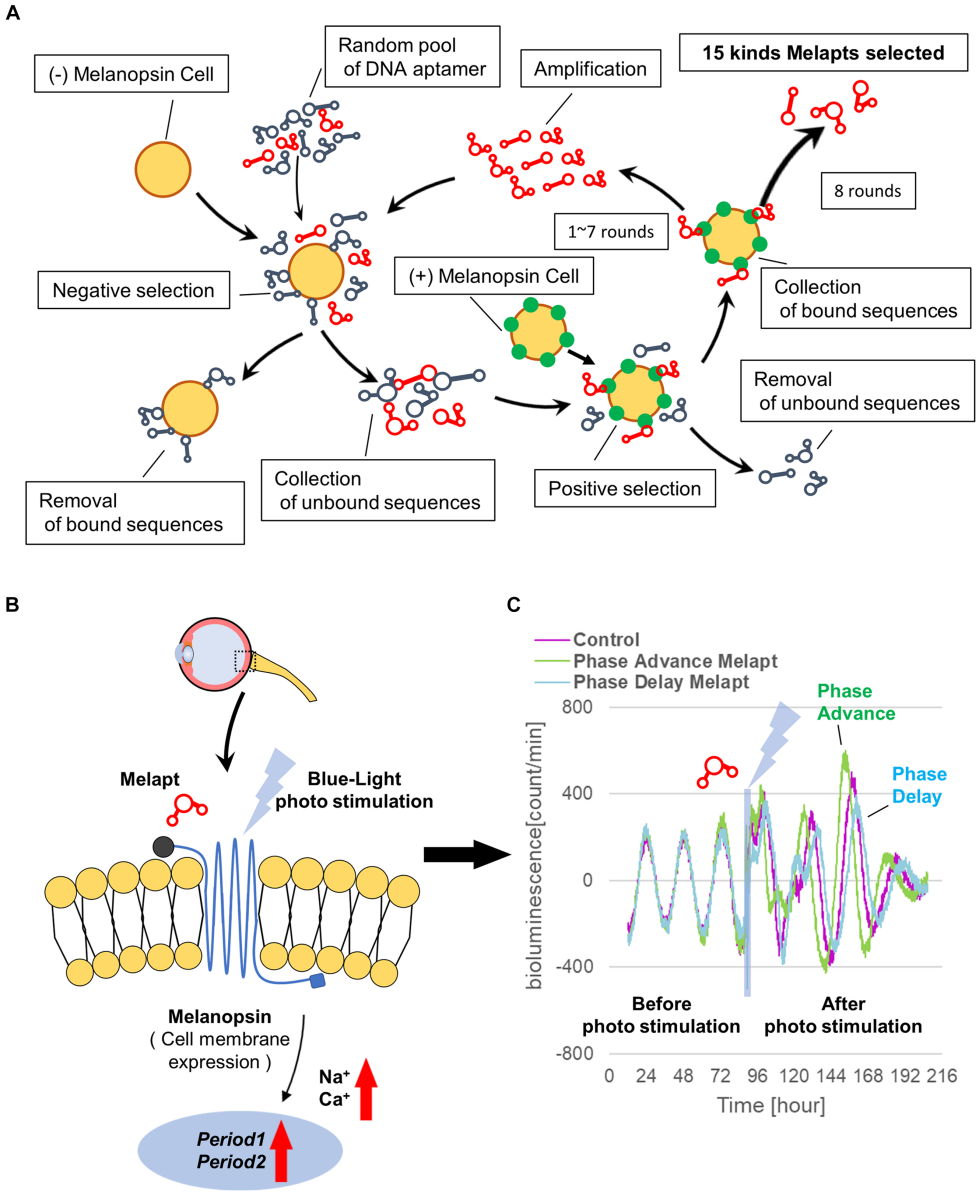
Cell-SELEX method for screening Melapts. Melapts that specifically bind melanopsin were selected by Cell-SELEX because melanopsin is a membrane protein bound and accessible from outside cells. **(A)** 10^17^ DNA aptamer were selected from a random ssDNA library. DNA aptamers were mixed and bound to (−) melanopsin cells (negative selection). When unbound DNA aptamers were retrieved, they were combined with (+) melanopsin cells to promote binding to melanopsin protein (positive selection). The Cell-SELEX process was performed over 8 rounds, and 15 types of Melapt were screened. Fluorescence intensity in the wells was measured (at λex 495 nm and λem 517 nm) using a microplate reader (Infinite 1,000; TECAN). **(B)** Conceptual diagram of Melapt binding to melanopsin followed by induction of the expression of the clock gene *Per2*. When melanopsin expressed on the surface of the cell membrane is photostimulated by a Melapt, the concentration of Na^+^ and Ca^+^ in the cytoplasm increases transiently, inducing the activation of transcriptional factors and the upregulation of *Per2* in the cell nucleus. **(C)** Phase shift of luciferase emission rhythms in *Per2:ELuc:*TK:Mel cells stably expressing melanopsin. When *Per2:ELuc* cells expressing melanopsin were photostimulated post-addition of Melapts, *Per2:ELuc* emission rhythms showed phase advance (green curve) or phase delay (blue curve) relative to controls (purple curve); these shifts were Melapt-dependent. PBS buffer alone was added to control cells.

A random ssDNA library for screening of DNA aptamers by the Cell-SELEX method was designed based on a random DNA aptamer (5‘-AAAGGGGAATTCGGATCC-N-40-CTGCAGAAGCTTCCGAAAA-3′) with regions of 40 bases, and 10^17^ types of DNA aptamers with a fixed leading and trailing region were prepared by Hokkaido System Science (Hokkaido, Japan). A floating PC12 cell (obtained from the Cell Bank, RIKEN BRC, Ibaraki, Japan) was used to perform the Cell-SELEX method, and cells were cultured in low-glucose DMEM (FUJIFILM Wako, Tokyo, Japan) containing 10% fetal bovine serum (FBS; Wako, Tokyo, Japan), 5% donor horse serum (DHS; Wako, Tokyo, Japan), and 1% penicillin–streptomycin (Wako, Tokyo, Japan). The melanopsin expression plasmid (provided by Professor Ueda of the University of Tokyo) was transfected into PC12 cells using Lipofectamine 3000 (Invitrogen, Massachusetts, United States) to prepare (+)melanopsin-transfected PC12 cells ([+] melanopsin cells) in which melanopsin was overexpressed on the cell membrane, whereas in (−) melanopsin-free PC12-negative control cells (other [−] melanopsin cells without transfecting melanopsin: negative selection), melanopsin was not expressed. First, DNA aptamers were mixed and incubated with (−) melanopsin cells, and only unbound DNA aptamers were recovered. Second, the recovered DNA aptamers in solution were mixed and incubated with (+) melanopsin cells, and only DNA aptamer bound to cells was recovered. Then, the recovered DNA aptamers specifically bound to (+) melanopsin cells but not bound to (−) melanopsin cells were amplified by asymmetric PCR amplofication (Figure 1A).

The obtained Melapts were amplified with forward and reverse primers in a 10:1 ratio by asymmetric PCR with reactions containing 10 pmol Melapt, 20 pmol forward primer (5′-AAAGGGGAATTCGGATCC-3′, FASMAC), 2 pmol reverse primer (5′-AAACGGAAGCTTCTGCAG-3′, FASMAC), 5 units Go Taq DNA polymerase (Promega; Wisconsin, USA), 30 nmol Mg^2+^ (Promega), 2.5 nmol dNTPs (Promega), and PCR buffer (Promega) in a final volume of 20 μL. The PCR amplification profile for Melapts involved preliminary denaturation at 95°C for 5 min, followed by 35 cycles of denaturation at 95°C for 30 s, annealing at 52°C for 1 min, extension at 72°C for 1 min, and a final extension at 72°C for 4 min.

Only those amplified as a significant sense band in gel electrophoresis were used in the next round of Cell-SELEX. These steps were repeated for eight rounds to concentrate DNA aptamers by asymmetric PCR amplification. Then, DNA aptamers were cloned into a T vector (Invitrogen), and aptamer sequences were confirmed by Fasmac Corporation (Kanagawa, Japan; Table 1). Finally, 15 DNA aptamers for melanopsin were selected and named Melapt1-Melapt15 (Supplementary Figure S1). Melapts obtained by the Cell-SELEX method appeared to bind specifically to melanopsin alone. Melanopsin-KO cells and melanopsin-KO mice were not used in this study.

**Table 1:** Sequences of melanopsin aptamers (Melapts).

Melapt01	CGACCCGAAGGAGCGGTGGATAACCCCACCAAGATACGTC
Melapt02	CCCGATTGGACAACTCAAACCATCATCCGAACCAGTACGT
Melapt03	GGCCGTCACGCCCCGGCTGCGAAGCCATCAAGCCTCCATA
Melapt04	CCGCACACGTGGAGCCAAGTCGAGTTTAATTCCCTATCGC
Melapt05	GAACTACCCTACAAACCAAACAAGGCGCACGATCGATATA
Melapt06	CCGATGTAGAAATCCAACACCGCAGTAGAGCATTGCCGAC
Melapt07	GAATCACTGGGCCCATGACCCCATGCAATACAAGAAGACT
Melapt08	TTCACCGATACGGCTCCCTTGGCCAGACAGGAAAAAATAA
Melapt09	AGCGTACGCCCAGGCCGGANTGGGACCGCAAACCCATTCG
Melapt10	GCCCCGGAGTGCGGCCTGAAAACCACCATCTATAAGCCAA
Melapt11	AGGATAATGAACCTTCGCCAGACCTACCCTAACAAGTCCCA
Melapt12	GAATTCCAGCACAGACCACCCTTGTCGAACCCAGCAACTCG
Melapt13	GAAGAGGGTGATCGTAATAACGCGTAAAACGAGACTATCT
Melapt14	GCACGGCGCGGTAGGCATGTCACTACCAGAAACTAGGCCC
Melapt15	CCCAAATCCATGAAAGGGGGAAACACAATCTTACGCCGCG

Sequences span regions of 40 bases. Melapts were generated by the Cell-SELEX method.

### Binding assay of Melapts to (+) melanopsin cells

**Figure 2:**
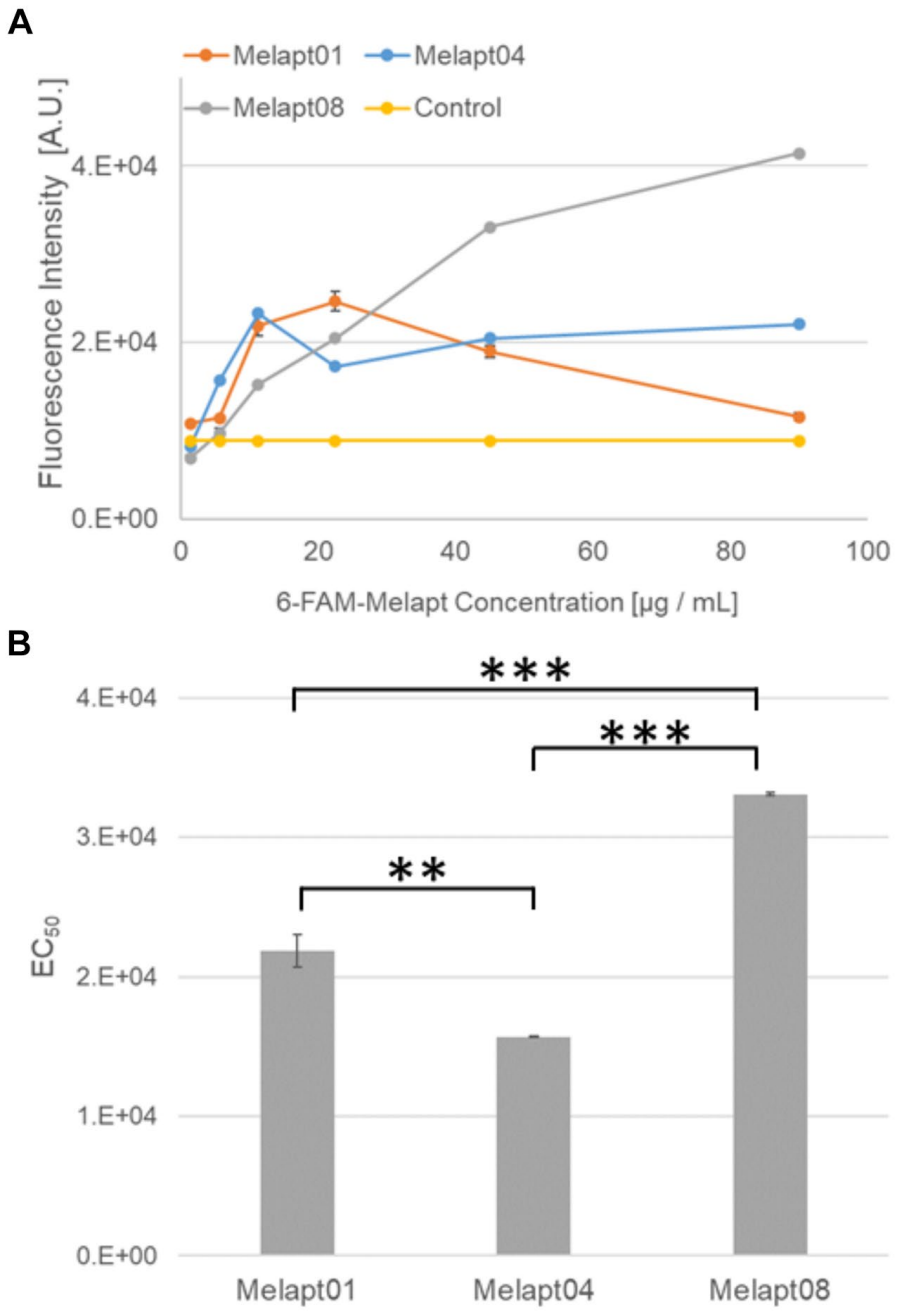
Binding of 6-FAM-Melapts to *Per2:ELuc:*TK:Mel stable cells expressing melanopsin. **(A)** 6-FAM-Melapts 01, 04, and 08 were added to cells at 1.405 μg/mL, 5.625 μg/mL, 11.25 μg/mL, 22.5 μg/mL, 45.0 μg/mL, and 90.0 μg/mL, whereas only PBS was added to control cells (*n* = 3). The 6-FAM fluorescence values of cells were plotted against the concentration of 6-FAM-Melapt. **(B)** The concentration resulting in EC_50_ was calculated from Figure 2A and plotted. Melapt01, 11.25 μg/mL; Melapt04, 5.625 μg/mL; Melapt08, 45.0 μg/mL. *n* = 3, **p < 0.01, ***p < 0.001, Tukey–Kramer test.

Melapts were amplified and labeled with a 6-FAM-forward primer. Briefly, 10 pmol Melapt, 20 pmol 6-FAM-forward primer (5′-AAAGGGGAATTCGGATCC-′3, Hokkaido System Science), 2 pmol reverse primer (5′-AAACGGAAGCTTCTGCAG-3′, Fasmac Corporation), 1 unit Go Taq DNA polymerase (Promega), 125 pmol dNTPs (Promega), 5× Go Taq Reaction Buffer (Promega), and nuclease-free water were combined in a 20 μL reaction volume using the Cell-SELEX amplification profile (Figure 2A).

Mouse fibroblast cells were cultured in 96-well plates (1.5 × 10^5^ cells/well) using 200 μL DMEM, transfected with melanopsin, and incubated at 37°C and 5% CO_2_ for 24 h. Then, 6-FAM-Melapt was added to the culture medium at six different concentrations (1.405 μg/mL, 5.625 μg/mL, 11.25 μg/mL, 22.5 μg/mL, 45.0 μg/mL, and 90.0 μg/mL) and incubated for 15 min. After washing, the binding capacity of 6-FAM-Melapts was estimated by measuring the fluorescence signal in (+) melanopsin cells (λex 495 nm, λem 517 nm) using an Infinite 1,000 microplate reader (Tecan, Zürich, Switzerland; Figure 1). The most suitable concentration of Melapts was 22.5 μg/mL. Before performing each measurement, 6-FAM-Melapt was added to three different wells (*n* = 3). A stable mouse fibroblast cell line (*Per2:ELuc:*TK:Mel) with Melapts was established to monitor the phase shift of *Per2:ELuc* bioluminescent emission rhythms using photo-responsive *ELuc*.

The 5′ flanking region of *Per2* (from −2,858 to +144, where +1 indicates the putative transcription start site) was PCR-amplified from the C57BL/6 J mouse genome and cloned into the *Xho*I and *Bgl*II sites of pELuc (PEST)-test (Toyobo, Osaka, Japan). Expression cassettes containing early poly-A (pA) signal, *Per2* promoter, ELuc-PEST, and late pA signal were amplified by PCR and cloned into pENTR-D-TOPO (Thermo Fisher Scientific Inc., Waltham, MA, United States), with the attL1 and attL2 sites flanked by the upstream and downstream regions of early and late pA signals, respectively, resulting in pm*Per2*-ELuc-PEST-pENTR. The expression cassette was recombined into the pBsd-R4 attB vector (a gift from Dr. T. Ohbayashi) by the LR reaction using LR Clonase II Plus Enzyme Mix (Thermo Fisher Scientific, Inc.), yielding pR4-Bsd-m*Per2*-ELuc-PEST.

Furthermore, mOpn4-Flag, wherein the Flag-tag was fused in-frame to the C-terminus of mouse melanopsin cDNA, was synthesized as double-stranded DNA (GenScript, Tokyo, Japan) and cloned into pUC57. The mOpn4-Flag was excised using *Nco*I and *Xba*I and ligated into the *Nco*I and *Xba*I site of pTK-SLG-pENTR-D-Topo (Tabei et al., 2017), from which the SLG cDNA was removed, yielding pTK-mOpn4-Flag-pENTR. The expression cassette containing TK promoter, mOpn4-Flag, and late pA signal was recombined into the pNeo-ϕC31 attB vector (Yamaguchi et al., 2011; a gift from Dr. T. Ohbayashi) by the LR reaction, resulting in pϕC31-Neo-mOpn4-Flag.

Mouse fibroblast A9 cells harboring the multi-integrase mouse artificial chromosome (MI-MAC) vector (Takiguchi et al., 2014; kindly provided by Dr. M. Oshimura and Dr. Y. Kazuki) seeded into six-well plates were co-transfected a day later with pR4-Bsd-m*Per2*-ELuc-PEST and the R4 integrase expression plasmid pCMV-R4 (Yamaguchi et al., 2011; kindly provided by Dr. T. Ohbayashi) and subcultured for selection with 6 μg/mL Blasticidin S (Thermo Fisher Scientific, Inc.). Selected cells were further co-transfected with pϕC31-Neo-mOpn4-Flag and ϕC31 integrase expression plasmid pCMV-ϕC31 (Yamaguchi et al., 2011; kindly provided by Dr. T. Ohbayashi) and subcultured for selection with 800 μg/mL G418 (Nacalai Tesque, Kyoto, Japan). Genomic PCR confirmed the integration of the transgenes into the corresponding sites in the MI-MAC vector. The established cell line was named *Per2:ELuc:*TK:Mel.

The photo-responsive fibroblast stable cell line for functional analysis of DNA aptamers (ELuc:*Per2:ELuc:*TK:Mel) was stably transfected into *Per2*-enhanced green-emitting luciferase cells (*Per2:ELuc*) with melanopsin (mOPN4) expression under the control of the TK promoter to generate photo-responsive A9 fibroblast cells (Nakajima et al., 2010). Screening of DNA aptamers was performed using blue light-responsive and bioluminescence real-time imaging of circadian rhythms (Supplementary Figure S2). *Per2:ELuc* stably expresses ELuc under the control of the *Per2* promoter because the phase due to the transcriptional activity rhythm of *Per2* can be monitored from the emission rhythms of ELuc (Nakajima et al., 2010). *Per2:ELuc:*TK:Mel stable cells constitutively and stably express melanopsin on the cell surface under the control of the TK promoter. Melanopsin expressed on the surface of the cell membrane transmits external photo-stimuli into the cell and transiently induces *Per2* transcription in the cell nucleus. Thus, *Per2:ELuc:*TK:Mel stable cells are suitable for the phase shift of circadian rhythms in response to blue-light photo-stimuli.

### Binding assay for Melapts

A total of 1.5 × 10^5^ cells/well were cultured in 96-well plates for 24 h using 200 μL of DMEM and incubated at 37°C and 5% CO_2_. 6-FAM-Melapt was added to (+) melanopsin cells at six different concentrations (1.405 μg/mL, 5.625 μg/mL, 11.25 μg/mL, 22.5 μg/mL, 45.0 μg/mL, and 90.0 μg/mL) and incubated for 15 min. After washing, the binding capacity of 6-FAM-Melapt in (+) melanopsin cells was estimated by measuring the 6-FAM signal using a microplate reader (λex 495 nm, λem 517 nm). Before performing each measurement, 6-FAM-Melapt was added to three different wells (*n* = 3).

### Estimation of phase shifts by *Per2: Eluc*:TK:Mel stable cells

Screening of DNA aptamers was performed using blue light-responsive and real-time bioluminescence recording of circadian rhythms (Supplementary Figure S2). *Per2:ELuc* cells were expressed in a stable cell line transfected to express ELuc under the control of the *Per2* promoter (Nakajima et al., 2010) because the phase due to the transcriptional activity rhythm of *Per2* can be monitored from the emission rhythms of ELuc. *Per2:ELuc:*TK:Mel stable cells expressed melanopsin stably under the control of the melanopsin under the control of the TK promoter. Melanopsin transmits external photo-stimuli into the cell via signal transduction and transiently induces *Per2* transcription in the cell nucleus. Thus, *Per2:ELuc:*TK:Mel stable cells were deemed suitable for the phase shift of circadian rhythms in response to blue-light photostimulation because of recombinant gene *Per2:ELuc* and TK:Mel.

*Per2: ELuc:* TK: Mel stable cells (1.5 × 10^5^) were passaged in a 35-mm dish, forskolin (Invitrogen) was added at a final concentration of 10 μM at 24 h after passaging, and cells were incubated for 30 min at 37°C before replacing the medium. This process synchronizes the circadian phase of *Per2* rhythmic expression in all cells to amplify the amplitude of the emission rhythm, monitored by Phot multiple tubes (PMTs). Following forskolin shock, the cells were washed with phosphate-buffered saline (PBS) and exposed to luciferin (Beetle Luciferin, Potassium Salt; Promega) at a final concentration of 0.1 mM in a new medium in a Kronos illuminometer (ATTO, Tokyo, Japan). This was equipped with a PMT to detect luminescence and measure the cell emission signal in dishes in real time for 10 s every 3 min, while the cells were incubated at 37°C with 5% CO_2_. The phase of *Per2:Eluc:*TK:Mel stable cells was determined as the middle point between the peak and trough of bioluminescent rhythms as CT12. Then, Melapts were added to the culture medium at 22.5 μg/mL (final concentration) at subjective dawn (CT22) and subjective afternoon (CT8). Photostimulation was then performed in culture dishes in the Kronos for 15 min using a Blue LED (Amon Industry Co., Ltd., Tokyo, Japan). Subsequently, rhythmic bioluminescence in *Per2:ELuc:*TK:Mel stable cells was observed for 3 days.

The obtained rhythmic emission data were analyzed by cosine fitting over a 12-36-h period after forskolin shock, and after photostimulation, using NINJA software (CHURITSU Electric, Aichi, Japan).

### Animals

*Per1::luc* Tg C57BL/N strain mice with a firefly luciferase gene linked to the downstream region of the 6.7-kb *Per1* promoter (provided by Professor Tei, Kanazawa University; Yamazaki et al., 2000) were used to monitor mammalian the circadian rhythm of the central clock in the SCN. Experiments were performed using *Per1::luc* Tg heterozygous mice. Mice (all male and aged 6 to 12 months) were maintained under SPF conditions at 22°C with a 12:12 h light:dark cycle (from 08:00 to 20:00). Mice were fed standard pellets (CLEA Rodent Diet CE-2, CLEA, Tokyo, Japan, Inc.), and water and food were freely available. All animals were handled according to the guidelines for the use of laboratory animals, Toyohashi University of Technology (DO2021-1).

### Injection of Melapts into bulbus oculi of *Per1::luc* Tg mice

Mice were injected with abatin anesthetic (1.9% w/v, 0.45 mL/20 g body weight). Response reflexes were assessed by pinching the tail and legs 5 min after anesthesia. For the mydriasis tests, one to two drops of Midorin-P ophthalmic solution (tropicamide phenylephrine hydrochloride ophthalmic solution; Santen Pharmaceutical, Osaka, Japan) were injected into the eyes of three Tg mice (CT22) following Melapt injection. Then, 1 μL of Melapt solution (100 ng-300 ng/μL) was injected into both bulbus oculi using a microsyringe (Hamilton, Nevada, United States) from the border between the cornea and sclera into both eyes. After injection, the mice were illuminated with an LED (Yazawa Corporation, Tokyo, Japan) at 1000 LUX for 30 min. Bulbus oculi were removed to prevent them from responding to light. All animal experiments were conducted under dim red LED lights.

### Observing circadian bioluminescence rhythms of SCN cultured slices

We prepared 300-μm coronal section slices of SCN using a micro-slicer (Dosaka E.M., Kyoto, Japan) in cooled PBS on ice from whole brains of three *Per1::luc* Tg mice following Melapt injection. Under a stereomicroscope, only the SCN on the Chiasma was removed as a 1.5-mm triangular tissue section from coronal section slices in 1.2 mL of media. The SCN tissue slices were grown on Millicell membranes (PICM03050; Millipore, Massachusetts, USA) in serum-free DMEM (Invitrogen) containing 10 mM HEPES (pH 7.2; Invitrogen), 2% B27 (Life Technologies, Carlsbad, CA, United States), 25 unit/mL penicillin, and 25 μg/mL streptomycin (Invitrogen) in a 35-mm dish. Beatle luciferin potassium salt (Promega) was added at 0.2 mM where required. Circadian bioluminescence rhythms were continuously monitored using a PMT (Hamamatsu Photonics) in an incubator at 36°C. Emission values of SCN slices were integrated for 1 min over ~5 days and plotted onto graphs. Circadian bioluminescence cycle data were examined using NINJA software.

### Statistical analysis

The Tukey–Kramer method in R software v.4.1.0. (R Foundation for Statistical Computing, Vienna, Austria) was used to analyze data. Statistical significance was set at *p* ≤ 0.05. Error bars on graphs show mean ± standard deviation (SD). All analyses were performed at least three times.

## Results

### Selecting DNA aptamers for melanopsin: Melapts

From the 10^17^ different DNA fragments, we used the Cell-SELEX approach to identify DNA aptamers that bind specifically to melanopsin in PC12 cells; eight rounds of Cell-SELEX were performed using 10^17^ DNA fragments, with (−) melanopsin cells as negative selection and (+) melanopsin cells as positive selection, respectively, by PCR to select 15 Melapts that bind specifically to melanopsin (Figure 1A). Functional screening of the 15 Melapts was performed to identify those able to induce a phase shift in circadian rhythms (Figure 1B). Photostimulation at subjective dawn (CT22) caused phase advance of *Per2* expression rhythms in *Per2:ELuc:*TK:Mel stable fibroblasts expressing melanopsin (Figure 1C, Tables 2, 3). In addition, some Melapts caused a more significant phase advance of circadian rhythms following photostimulation (Figure 1B). These findings suggest that Melapts influence the phase of circadian rhythms by binding to melanopsin, thereby triggering signal transmission into cells and affecting transcription of the clock gene *Per2*.

**Table 2:** Summary of phase shifts induced by Melapts *in vitro* and *in vivo.*

*In vitro*					*In vivo*
Melapt_No.	CT22_BL	CT8_BL	CT22_nonBL	CT8_nonBL	CT22_BL
Control	1.3	−1.0	−0.6	−0.1	0.0
Melapts 1	8.6	9.8	−6.0	−0.1	0.5
Melapt02	7.0	1.3	0.9	−3.5	−0.7
Melapts 3	6.4	1.1	−3.4	−3.1	1.6
Melapts 4	6.0	0.3	7.4	4.4	3.1
Melapt05	1.0	−2.4	−4.8	9.4	ND
Melapt06	−0.4	2.5	6.6	7.7	ND
Melapt07	−1.0	1.3	−10.4	3.1	−1.1
Melapt08	−1.2	6.7	−7.8	1.1	ND
Melapt09	−4.4	7.3	3.0	8.1	−0.7
Melapts 10	−4.6	−1.1	−5.7	−6.3	−2.0
Melapt11	−5.5	0.4	1.0	4.0	0.3
Melapt12	−5.5	5.4	−9.1	4.7	ND
Melapts 13	−5.8	−6.4	5.2	8.3	ND
Melapt14	−8.4	0.5	1.9	1.1	ND
Melapt15	−4.8	4.4	5.2	−3.9	ND
					/h

Summary of phase shifts in circadian rhythms induced by each Melapt in *Per2: Eluc*:TK:Mel stable cells and in SCN of *Per1::luc* Tg mice. Positive and negative numbers indicate phase delay and advance (h), respectively. Melapts 1,3,13 described in red induced phase advance or delay in the absence of blue light, while Melapts described in black induced reverse phase delay or advance in the presence of blue light. Melapts 4,10 described in blue induced phase shifts in both the presence and absence of blue light. In total, six of nine Melapts displayed similar phase shifts at CT22 *in vitro* and *in vivo* in the absence of blue light.

**Table 3:** Summary of phase shifts induced *in vitro* and *in vivo.*

*In vitro*							
Melapt_No.	CT22_BL	Melapt_No.	CT8_BL	Melapt_No.	CT22_nonBL	Melapt_No.	CT8_nonBL
Melapt01	8.6	Melapt01	9.8	Melapt04	7.4	Melapt05	9.4
Melapt02	7.0	Melapt09	7.3	Melapt06	6.6	Melapt13	8.3
Melapt03	6.4	Melapt08	6.7	Melapt13	5.2	Melapt09	8.1
Melapt04	6.0	Melapt12	5.4	Melapt15	5.2	Melapt06	7.7
Control	1.3	Melapt15	4.4	Melapt09	3.0	Melapt12	4.7
Melapt05	1.0	Melapt06	2.5	Melapt14	1.9	Melapt04	4.4
Melapt06	−0.4	Melapt02	1.3	Melapt11	1.0	Melapt11	4.0
Melapt07	−1.0	Melapt07	1.3	Melapt02	0.9	Melapt07	3.1
Melapt08	−1.2	Melapt03	1.1	Control	−0.6	Melapt14	1.1
Melapt09	−4.4	Melapt14	0.5	Melapt03	−3.4	Melapt08	1.1
Melapt10	−4.6	Melapt11	0.4	Melapt05	−4.8	Control	−0.1
Melapt15	−4.8	Melapt04	0.3	Melapt10	−5.7	Melapt01	−0.1
Melapt11	−5.5	Control	−1.0	Melapt01	−6.0	Melapt03	−3.1
Melapt12	−5.5	Melapt10	−1.1	Melapt08	−7.8	Melapt02	−3.5
Melapt13	−5.8	Melapt05	−2.4	Melapt12	−9.1	Melapt15	−3.9
Melapt14	−8.4	Melapt13	−6.4	Melapt07	−10.4	Melapt10	−6.3
							/h
*In vivo*							
Melapt_No.				CT22_BL			
Melapt04				3.1			
Melapt03				1.6			
Melapt01				0.5			
Melapt11				0.3			
Control				0.0			
Melapt02				−0.7			
Melapt09				−0.7			
Melapt07				−1.1			
Melapt10				−2.0			

Summary of the phase shift in circadian rhythms induced by each Melapt in *Per2: Eluc*:TK:Mel stable cells and in the SCN of *Per1::luc* Tg mice. Positive and negative numbers indicate phase delay or advance (hours), respectively. Melapts highlighted in orange and blue lines induce the same phase shifts described in the footnote to Table 2.

### Estimating the binding capacity of Melapts

We selected three Melapts labeled with 6-FAM (Melapt01, Melapt04, and Melapt08), Melapt01 (group1) induced both phase advance of *Per2::luc* bioluminescence rhythms and rhythm phase delay at CT22 (Figure 2).

That was because15 screened Melapts were divided into three groups, group 1: phase advance or delay in *Per2:ELuc* bioluminescence rhythms at both CT22 and CT8 like Melapt04, group 2: phase advance or delay at CT22 and delay or advance at CT8, respectively, like Melapt01, and group 3: non-phase shifts in *Per2:ELuc* bioluminescence rhythms like Melapt08. Melapt01, Melapt04, and Melapt08 were selected as representatives of each group and were FAM-labeled to determine a final concentration of Melapt for adding on cells. At 22.5 μg/mL, all three Melapts could bind to melanopsin on the cell membrane sufficiently.

Three Melapts labeled with 6-FAM fluorescence using 6-FAM primers were compared in terms of binding capacity to *Per2:ELuc:*TK:Mel stable cells. The higher binding capacity of Melapt04 and Melapt01 to cells was saturated at a concentration of 11.25 μg/mL, whereas that of Melapt08 could not be saturated at a concentration of 90 μg/mL (Figure 2A). Thus, in subsequent experiments, the applied concentration of Melapts was 22.5 μg/mL, sufficient to bind to melanopsin on the cell membrane because the half-maximal effective concentration (EC_50_) of Melapt08 with even lower binding capacity was 22.5 μg/mL (Figure 2B). These results not only confirmed the successful screening of selective aptamers capable of binding to melanopsin cell membranes but also determined the appropriate concentrations of Melapts in the culture medium for phase-shift experiments.

### Screening of Melapts using *Per2: Eluc*:TK:Mel stable cells

#### Melapt with blue-light photostimulation at CT22

The 15 Melapts were categorized according to their ability to trigger a phase shift in circadian rhythms (Table 2). In addition, Table 3 shows Melapts rearranged from phase advance (positive number) to phase delay (negative number). The phase shift in bioluminescence rhythm depends on the timing of Melapt addition and the presence of photostimulation.

**Figure 3:**
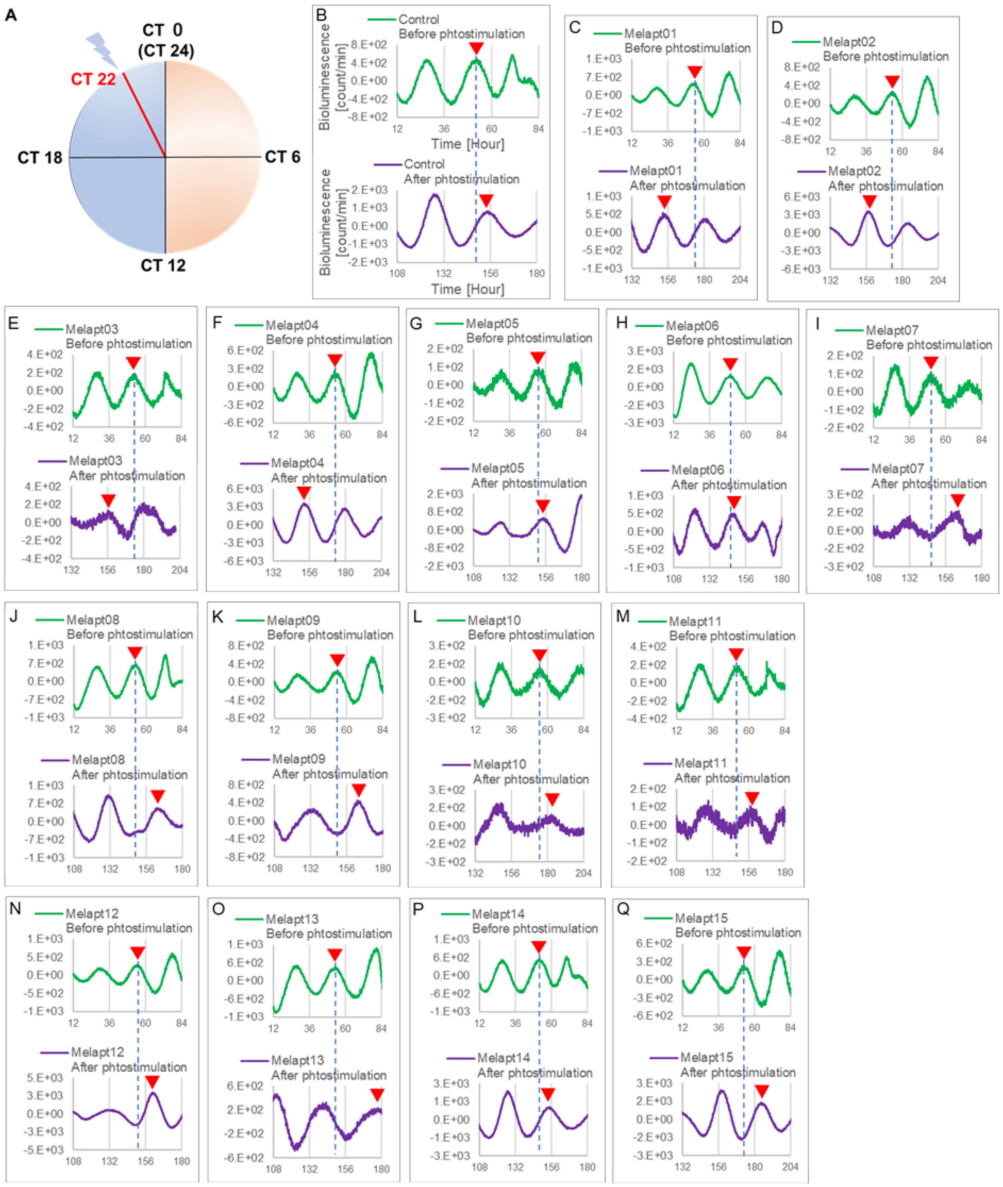
Changes in *Per2:ELuc* emission rhythms before and after the addition of each Melapt and blue-light photostimulation at CT22. **(A)** Subjective light period from CT0-12, subjective dark period from CT12-0, and a blue light indicate photostimulation at CT22 of the 24-h clock. **(B)** *Per2:ELuc* emission upon adding PBS and blue-light photostimulation as a control. **(C–Q)**. *Per2:ELuc* emission upon adding Melapt01-Melapt15 and blue-light photostimulation. Upper row (green), *Per2:ELuc* emission before the addition of Melapt observed for 3 days. Lower row (purple), *Per2:ELuc* emission after addition of Melapt and blue-light photostimulation observed for 3 days. A red triangle indicates the peak. Bioluminescence traces in Figure 3 were estimated from an individual sample. The peak bioluminescent emission rhythms monitored over 3 days before the addition of Melapts and photostimulation (green) are denoted by a dotted line to allow comparison with the peak rhythms in the lower graphs (plotted post-stimulation and colored purple).

*Per2:ELuc:*TK:Mel cells stably expressing melanopsin were exposed to each Melapt under blue-light photostimulation at CT22 as a model for exposure of the retina to intense blue light at dawn (Figure 3; Tables 2, 3).

**Figure 4:**
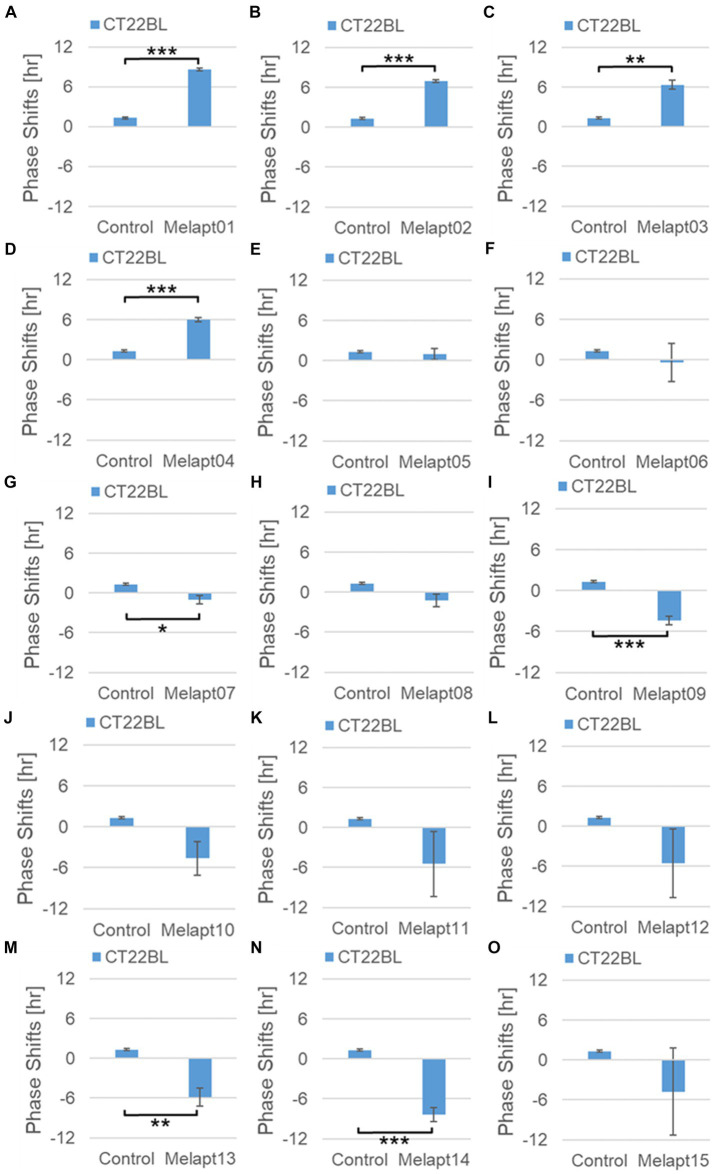
Phase shift due to binding of Melapts plus photostimulation at subjective dawn (CT22). **(A–O)** Phase-shifts comparison between Melapts and controls with photo-stimulus at CT22. PBS alone was added to control cells. The phase shift was calculated from the cosine-fitting curve in Figure 3 using the NINJA program and plotted on a graph. *n* = 3 (individual samples); *p < 0.05, **p < 0.01, ***p < 0.001, Tukey–Kramer test.

Phase advance of *Per2:ELuc* rhythmic emission rhythms by ~1.3 h was observed (using a Kronos bio luminometer) in controls following simple blue-light photostimulation in the absence of Melapts at CT22 (Figure 4; Tables 2, 3). Cells treated with Melapt01 showed a phase advance of *Per2:ELuc* emission rhythms by ~8.6 h (Figure 4A). The group treated with Melapt02 showed a phase advance of *Per2:ELuc* emission rhythms by ~6.9 h (Figures 4B–E). Melapt05 induced a phase advance of *Per2:ELuc* emission rhythms by ~1.0 h (Figure 4E). Conversely, Melapt06 induced a phase delay of *Per2:ELuc* emission rhythms of ~0.4 h (Figure 4F). Groups treated with Melapt07, Melapt08, Melapt09, Melapt10, Melapt11, Melapt12, Melapt13, Melapt14, and Melapt15 showed phase delays of *Per2:ELuc* emission rhythms by approximately 1.0, 1.2, 4.3, 4.6, 5.4, 5.5, 5.8, 8.4, and 4.7 h, respectively (Figures 4G–O).

#### Melapt with blue-light photostimulation at CT8

**Figure 5:**
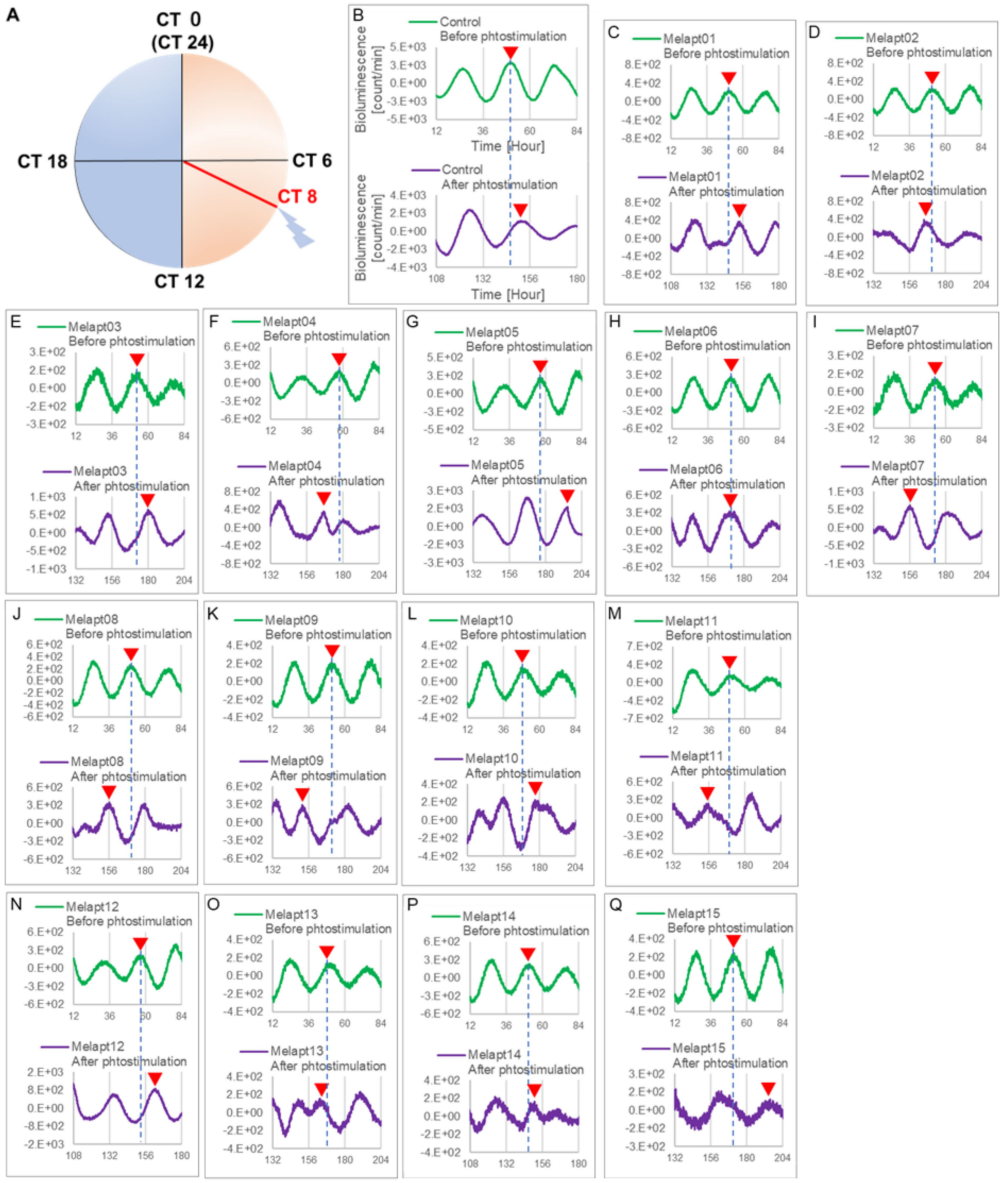
Changes in *Per2:ELuc* emission rhythms before and after applying Melapts and blue-light photostimulation at CT8. **(A)** Subjective light period from CT0-12, subjective dark period from CT12-0, and blue light indicate photostimulation at CT8 of the 24-h clock. **(B)** *Per2:ELuc* emission upon applying PBS and blue-light photostimulation to controls. **(C–Q)** *Per2:ELuc* emission upon applying Melapt01- Melapt15 and blue-light photostimulation. Upper row (green), *Per2:ELuc* emission before the addition of Melapt observed for 3 days. Lower row (purple), *Per2:ELuc* emission after addition of Melapt and blue-light photostimulation observed for 3 days. Red triangles indicate the peak. Bioluminescence traces in Figure 5 were estimated from an individual sample. The peak of bioluminescent emission rhythms measured over the 3 days prior to the addition of Melapts and photostimulation (green) is denoted by a dotted line to allow comparison with peak rhythms in the lower graphs (potted post-stimulation).

Then, we examined the effects on *Per2:ELuc* emission rhythms of applying Melapts and blue-light photostimulation in the subjective afternoon (CT8) to mimic exposure to intense afternoon light. Kronos bio luminometer results revealed that controls receiving only blue-light photostimulation without any Melapts at CT8 showed a phase delay of *Per2:ELuc* emission rhythms by ~0.6 h (Figure 5; Tables 2, 3).

**Figure 6:**
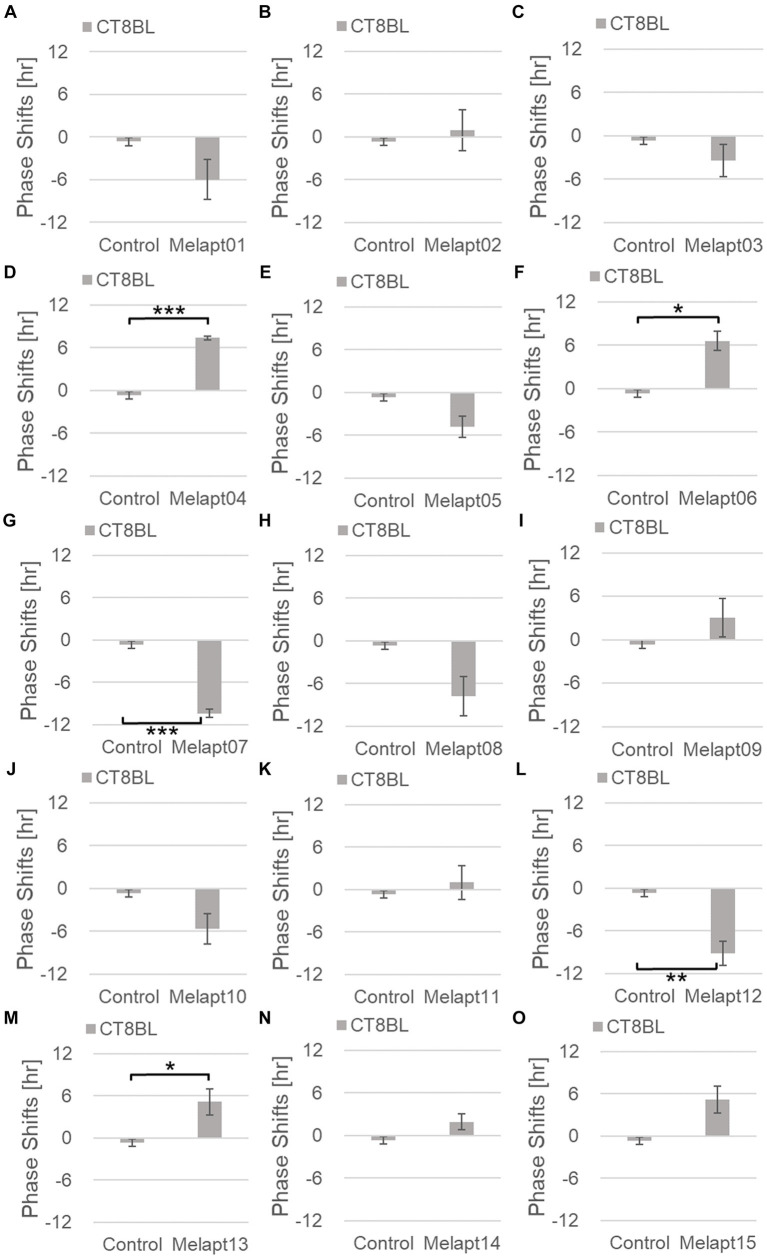
Phase shifts upon binding of Melapts plus photostimulation at subjective afternoon (CT8). **(A–O)** Phase-shifts comparison between Melapt-treated groups and controls with photo-stimulus at CT8. PBS alone was added to the controls. *n* = 3, *p < 0.05, **p < 0.01, ***p < 0.001, Tukey–Kramer test.

Cells treated with Melapt01 showed a phase delay of *Per2:ELuc* emission rhythms by ~5.9 h (Figure 6A). The Melapt03, Melapt05, Melapt07, Melapt08, Melapt10, and Melapt12 groups showed a phase delay of *Per2:ELuc* emission rhythms by approximately 3.4, 4.8, 10.4, 7.8, 5.6, and 9.1 h, respectively (Figures 6C,E,G,H,J,L). Melapt09 triggered a phase advance of *Per2:ELuc* emission rhythms by approximately 3.0 h (Figure 6I). By contrast, Melapt06 induced a phase delay of *Per2:ELuc* emission rhythms by ~6.6 h (Figure 6F). Groups treated with Melapt02, Melapt04, Melapt11, Melapt13, and Melapt14 displayed a phase advance of *Per2:ELuc* emission rhythms by approximately 0.9, 7.3, 1.0, 8.3, and 1.9 h, respectively (Figures 6B,D,K,M,N). Melapt15 induced a phase advance of *Per2:ELuc* emission rhythms by ~5.1 h (Figure 6O).

#### Melapt with and without blue-light photostimulation at CT22 and CT8

**Figure 7:**
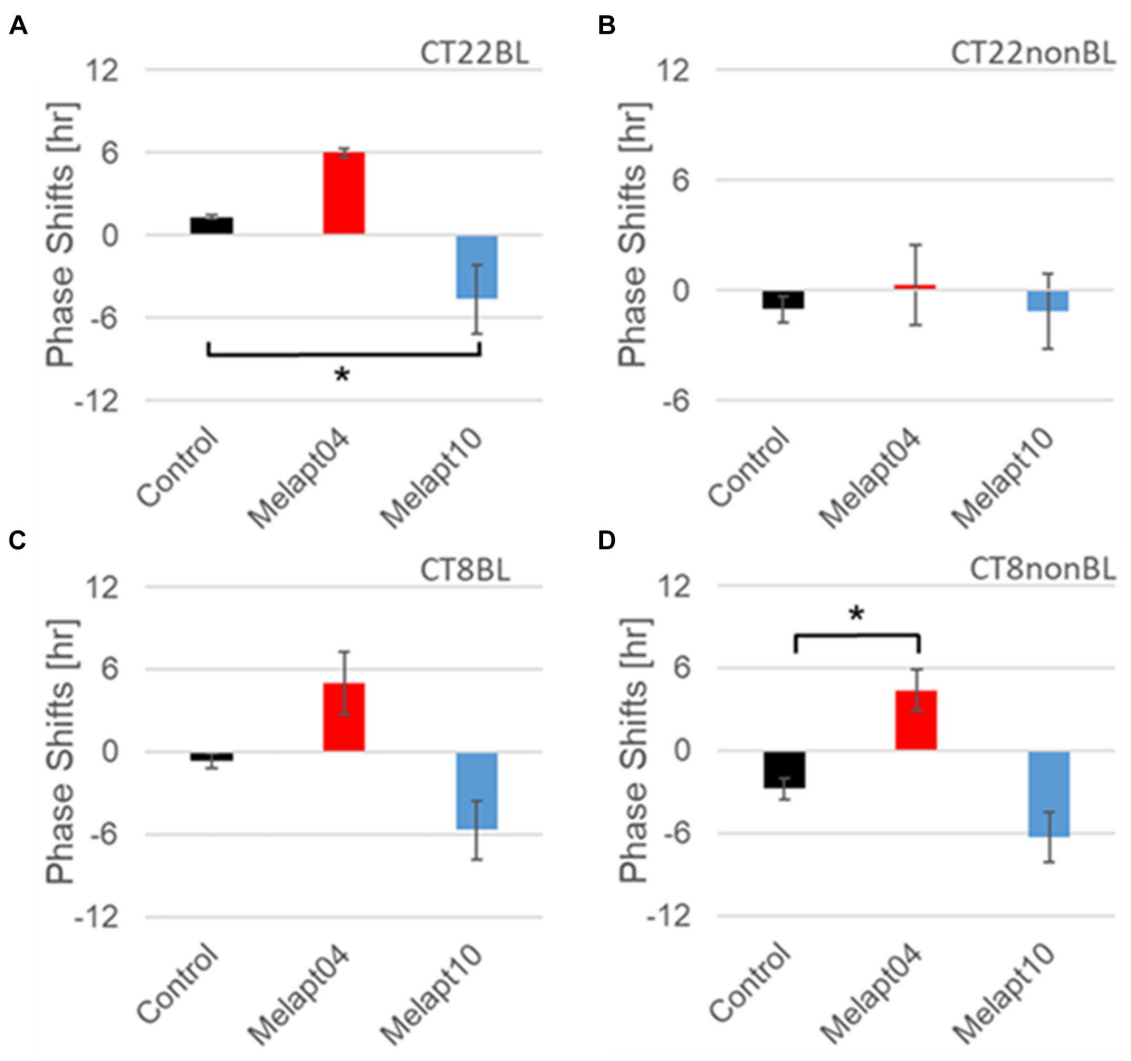
Similar phase-shift abilities of representative Melapts at both CT22 and CT8. **(A)** Melapt binding-mediated phase shift induced via photostimulation at subjective dawn (CT22BL). **(B)** Melapt-mediated phase shift at subjective dawn (CT22nonBL). **(C)** Melapt binding-mediated phase shifts induced via photostimulation in the afternoon (CT8BL). **(D)** Melapt-mediated phase shifts in the afternoon (CT8nonBL). Controls showed phase shifts in the absence of Melapts under all conditions. The upper direction shows phase advance, whereas the lower direction shows phase delay. Red: phase advance. Blue: phase delay. *p < 0.05, Tukey–Kramer test.

**Figure 8:**
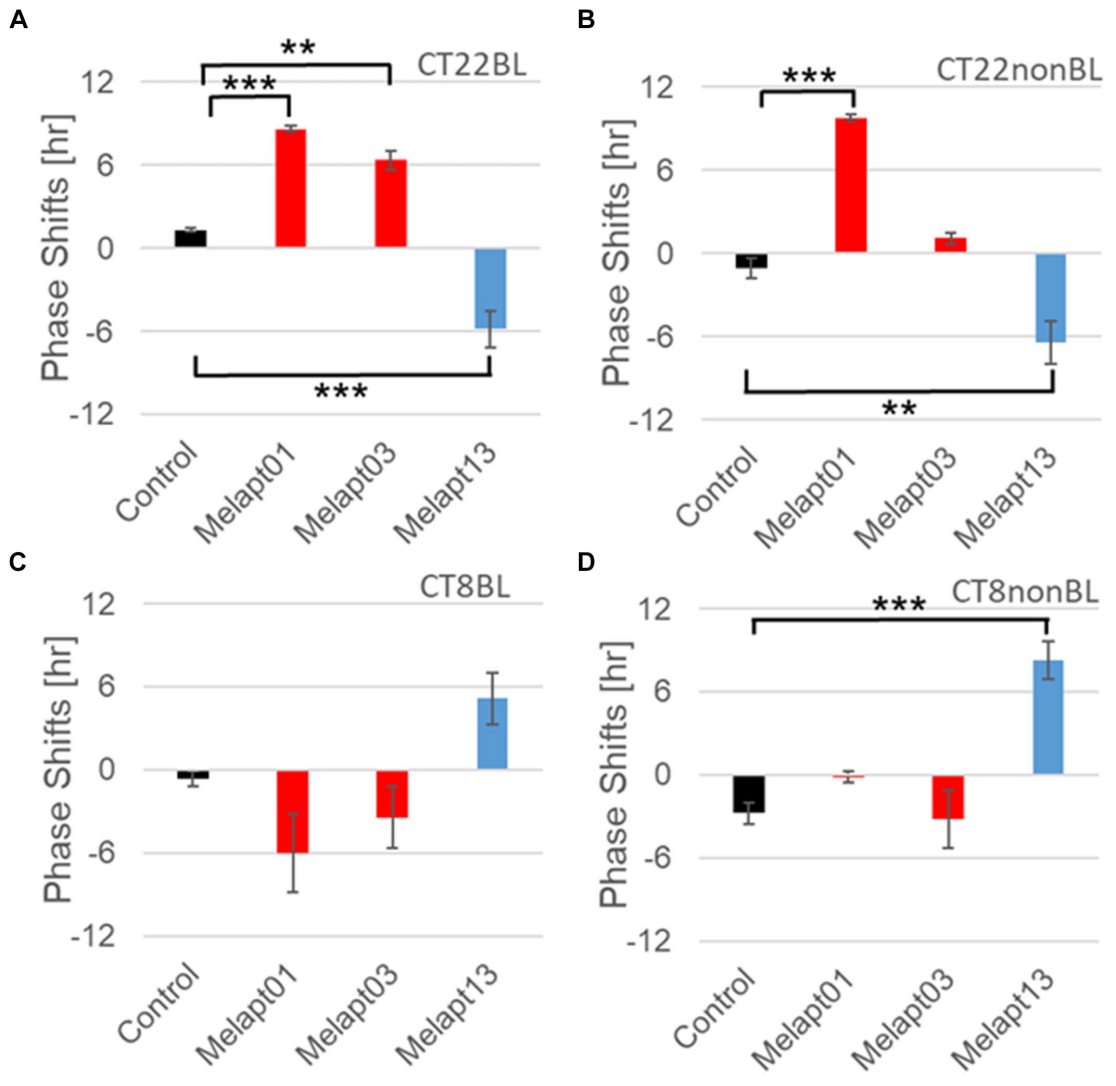
Reverse-phase shift abilities of representative Melapts at CT22 and CT8. **(A)** Melapt-mediated phase shift induced by photostimulation at subjective dawn (CT22BL). **(B)** Melapt-mediated phase shift at subjective dawn (CT22nonBL). **(C)** Melapt-mediated phase shift induced by photostimulation in the afternoon (CT8BL). **(D)** Melapt-mediated phase shift in the afternoon (CT8nonBL). Controls were phase shifts without any Melapts under all conditions. The upper direction shows phase advance, whereas the lower direction shows phase delay. Red: phase advance. Blue: phase delay. **p < 0.01, ***p < 0.001, Tukey–Kramer Test.

At CT22, Melapt01, Melapt03, and Melapt04 plus photostimulation induced a phase advance, while Melapt10 and Melapt13 at CT22 induced a phase delay (Figures 7A, 8A). We picked up Melapt4 and Melapt10 as representatives of Melapts in group1, which induced a phase advance and phase delay of *Per2:ELuc* bioluminescence rhythms in the same direction at both CT22 and CT8 (Figure 7). Melapt1, Melapt3, and Melapt13 were selected as representatives of Melapts in group2, which induced those of *Per2:ELuc* rhythms in opposite direction at CT22 and CT8 (Figure 8), for example, phase advance at both CT22 and CT8 by Melapt4, while phase advance at CT22 and phase delay at CT8 by Melapt1.

Although these phase shifts could be observed even without photostimulation when adding Melapts, Melapt04 and Melapt10 enhanced the phase shift compared with controls without photostimulation at CT22 (Figures 7B, 8B).

The phase-shift abilities of Melapts without photostimulation were evaluated (Figures 7B, 8B).

Melapts plus intense photostimulation in the subjective afternoon (CT8) caused phase shifts of *Per2* emission rhythms in the same direction (advance or delay) as at dawn (CT22; Figures 7A,C). In addition, at CT22, the degree of phase shift was changed more than at CT8 in both advanced and delayed directions. For Melapt01 and Melapt13, a more significant phase advance or delay at CT22 was observed in the absence of photostimulation. By contrast, for Melapt03, Melapt04, Melapt10, and Melapt13, a more significant phase advance or delay at CT8 was observed in the absence of photostimulation.

Furthermore, Melapt01 and Melapt03 advanced the phase at CT22, and Melapt13 delayed the Phase, while the opposite phase shifts were observed at CT8 (Figures 8A,C). Like Melapt04, Melapt01 and Melapt03 induced expression of *Per2* by binding to melanopsin at CT22, probably resulting in a phase advance, whereas they caused a phase delay at CT8.

Melapt03 induced a phase advance and delay at CT22 and CT8, while Melapt04 induced a phase advance at both CT22 and CT8. Nevertheless, the phase shifts of Melapt03 and Melapt04 without blue-light photostimulation were less than those with photostimulation at CT22, when *Per2* transcription was slightly upregulated (Figures 7A,B, 8A,B). However, phase shifts of Melapt03 and Melapt04 were the same with and without blue-light photostimulation at CT8, when *Per2* transcription was shown to be high (Figures 7A,B, 8A,B). Melapt04 and Melapt10 displayed little difference in phase-shift ability between CT22 and CT8 upon photostimulation (Figures 7A,C).

### Functional analysis of Melapts in *Per1::Luc* Tg mice

**Figure 9:**
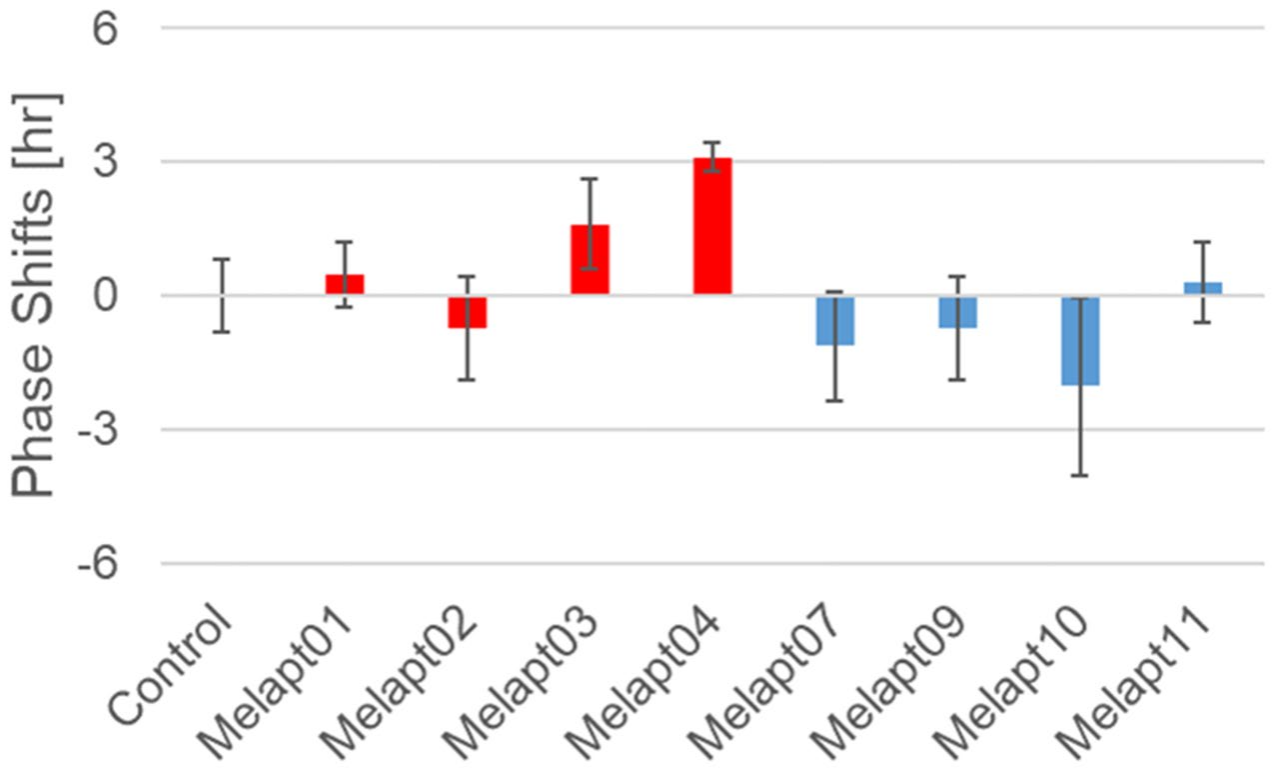
Phase shift of *Per1::luc* expressional rhythms in SCN slices following injection of Melapts into both bulbus oculi of *Per1::luc* Tg mice. Phase shifts following injection of Melapts into bulbus oculi with LED light stimulus at subjective dawn (CT22). SCN slices were obtained from mice injected with Melapts, and bioluminescence was observed for approximately 5 days. The NINJA program was used to calculate phase shifts. The upper direction shows phase advance, whereas the lower direction shows phase delay. Red: phase advance. Blue: phase delay. *n* = 3, *p < 0.05, Tukey–Kramer test.

We performed *in vivo* experiments similar to the *in vitro* experiments to investigate whether Melapts binding to melanopsin in the retina projecting to the SCN affected the phase shifts of the central clock in the SCN. Eight types of Melapt causing phase-shift responses in *Per2* expression rhythms in *in vitro* experiments were injected into the bulbs of eyes of *Per1::luc* Tg mice at CT22 (Figure 9; Tables 2, 3; Supplementary Figure S3). Melapt01, Melapt03, Melapt04, Melapt07, Melapt09, and Melapt10 displayed similar phase-shift abilities in the *in vivo* and *in vitro* experiments. Figures 4, 9 show that Melapt01, Melapt03, and Melapt04 induce phase advance and that Melapt07, Melapt09, and Melapt10 induce phase delay in CT22 both *in vivo* and *in vitro* as Melapts effect on phase shift by *in vivo* experiments can be predicted from *in vitro* experiments. On the other hand, total phase shifts were limited to 3 h in intact animals, regardless of how much advance or delay by Melapts in *Per2:Eluk:TK:Mel* cells.

For example, Melapt04 caused a maximum phase advance of 3 h, and Melapt10 caused a maximum phase delay at 3 h, in experiments in *Per2:ELuc:*TK:Mel stable cells and in SCN of *Per1::luc* Tg mice similar to the restriction of phase shifts to a maximum of 3–4 h in mammals (Gillette and Mitchell, 2002; Guler et al., 2008; Iyer et al., 2014; Figure 9; Tables 2, 3).

In total, two/three out of the nine Melapts showed similar phase shifts at CT22 *in vitro* and *in vivo* in the absence of blue light (Tables 2, 3). This suggests that each Melapt induced a similar phase shift in both mice and Per2:*ELuc*:TK:Mel stable cells.

## Discussion

### Melapts were identified to affect the phase of the circadian rhythms

This study focused on controlling the ability of melanopsin to manipulate the phase resetting of circadian rhythms caused by blue-light input from the eyes using Melapts identified by screening and *Per2*:*ELuc*:TK:Mel stable cells for functional analysis of phase-shift ability in molecular clock rhythms. Because we used cells exhibiting stable expression of melanopsin via the TK promoter, whereas previous studies (Pulivarthy et al., 2007; Ukai et al., 2007) used cells transiently transfected with melanopsin, we were able to perform more quantitative functional analyses of the phase response to aptamer concentration and light intensity.

Among the 15 Melapts obtained by Cell-SELEX, two showed characteristic responses: Melapt04 caused a phase advance and Melapt10 caused a phase delay at both subjective dawn (CT22) and in the afternoon (CT8). At dawn (CT22), when transcript levels of clock gene *Per2* began to increase, Melapt04, Melapt01, and Melapt03 increased signaling into cells to upregulate *Per2* transcription and induce a phase advance, while Melapt10 and Melapt13 repressed intracellular signaling to downregulate *Per2* expression and induce a phase delay. In the afternoon (CT8), *Per2* transcription was almost at its peak. It appeared that Melapt04 and Melapt10 with photostimulation caused the same phase shift as at CT22 and that Melapt1, Melapt3, and Melapt13 with photostimulation caused the opposite phase shift to that at CT22. At CT8, the transition between stopping the upregulation of *Per2* transcription and beginning its downregulation could account for the instability effect of the phase shift upon adding Melapt. Furthermore, we found that in both the presence/absence of blue light, the phase of the molecular clock can be shifted (either advanced or delayed) simply by the addition of a Melapt in contrast with previous reports (Takumi et al., 1998; Pulivarthy et al., 2007; Ukai et al., 2007).

Photostimulation by blue-light exposure of the retina of mice before dawn (CT22) resulted in a phase advance of activity rhythms, similar to previous reports in rats (Honma et al., 1985). However, adding a particular Melapt (01, 03, 04) at CT22 resulted in a more significant phase advance, whereas other Melapts (10 and 13) resulted in a phase delay.

At CT22 (subjective dawn), as *Per2* expression levels were increasing, the melanopsin signal bound to Melapt01, Melapt03, and Melapt04 with photostimulation had a complementary effect and enhanced *Per2* expression, although the detailed underlying mechanism is not known. The extent of the phase advance was higher than in controls only receiving photostimulation at the same time.

By contrast, among the phase delay group of Melapts, Melapt10 and Melapt13 at CT22 could be binding to melanopsin, leading to upregulation of *Per2* expression and repression of signal transduction in cells, and induction of *Per2* gene expression in cells. Therefore, the delay of PER2 protein accumulation in cells could lead to a phase delay of intracellular circadian rhythms.

Melapts binding to melanopsin could directly or indirectly modify the structure of the domain in melanopsin that binds to the retinal, altering the phase shifts. Melanopsin loses its photosensitivity when its structure, function, and retinoid cycle are altered (Dua et al., 2011; Harrison et al., 2021). For example, *cis-*retinal is present in cells bound to melanopsin without post-stimulation, which mediates photostimulation signal transduction into cells, as Melapts bound to melanopsin may influence intracellular signaling and phase shifts without photostimulation.

The secondary structure of Melapts was assessed (not shown) using GENETYX Ver. 9 (GENETYX CORPORATION). Melapt02 and Melapt04 in the phase advance group have an outwardly protruding C-A-G-A-G sequence within the secondary structure of the stem and loop. The stem–loop conformation may enhance the binding of Melapts to melanopsin. However, it is unclear whether Melapts directly or indirectly alter their binding domain to affect signal transfer or their ability to phase-shift circadian rhythms.

Both *per1* and *per2* are clock genes involved in an input pathway, and their transcription is induced transiently by photosignals, resulting in a phase shift in rhythmic transcription (Shigeyoshi et al., 1997; Yan et al., 1999; Ikegami et al., 2020). Therefore, the phase shift of *per1::luc* and *per2::luc* rhythm induced by Melapts is synchronized. We confirmed whether the aptamers obtained by Selex screening and functionally analyzed using a *per2::luc* reporter cells *in vitro* could trigger the same phase shift in the SCN of *per1::luc* Tg mice *in vivo*. We identified an agonist DNA aptamer that caused a maximum 8-h shift *in vitro* and a 3-h shift *in vivo*. There was a 3-h phase-shift limit *in vivo* because the photic input signal by melanopsin is transferred to the SCN projected from retinal ganglion cells by synaptic transmission (Hanna et al., 2017).

The 11 *cis*-retinal bound to melanopsin is isomerized to *trans*-retinal using blue light (485 nm), causing its dissociation from melanopsin (Schwartz and Zimmerman, 1990). Compared with photostimulation, Melapts that induced a phase advance (Melapt03 and Melapt04) and a phase delay (Melapt10) displayed lower phase-shift abilities, possibly because most retinal molecules remained in the *cis* isomeric form and therefore did not dissociate from melanopsin and transmit signals into cells, thereby repressing the phase shift. This signal of melanopsin is transmitted inside the cell via G protein-coupled receptors, eventually upregulating transcription of the clock gene *Per2* in cells (Iyer et al., 2014; Krzysztynska-Kuleta et al., 2021), resulting in a phase shift of circadian rhythms. Further studies are needed to understand how the downregulation/upregulation of *Per2* upon Melapt–melanopsin binding induces phase shifts of circadian rhythms via CREB phosphorylation.

### Future research on Melapt based regulation of the intracellular clock

Melapt04 and Melapt10 induced phase advance or delay of the circadian clock by ~3 h, respectively, at both CT22 and CT8 during the photo-signal input process. This suggests that Melapt04 regulates the phase of circadian rhythms, and facilitates falling asleep and waking, mainly via Phase Advance. Considering the social desire to advance the phase of the circadian clock to facilitate going to bed earlier at night and waking up earlier in the morning, Melapts could be used to trigger phase advance in humans. We chose to use DNA aptamers because they can be synthesized easily by PCR and are stable in the bloodstream. However, delivering aptamers to the retina is very difficult in humans (Hosoya and Tomi, 2005; Del Amo et al., 2017; Ikegami et al., 2020; Ham et al., 2023); here, we injected aptamers intraocularly into mice. Therefore, we need to develop a method for introducing aptamers efficiently and stably into the retina, possibly using a cell-permeable drug delivery with functions like ribosomes administered via drops into the eye (Wang et al., 2010; An et al., 2019; Yan et al., 2019; Tawfik et al., 2022).

Melapts could be used to regulate the function of membrane proteins by inducing conformational changes, thereby allowing more complex functional switching of the target (i.e., melanopsin) in the presence or absence of external photo-stimuli (Faria and Ulrich, 2002; Ulrich, 2005; Wang et al., 2010; Jones et al., 2013). However, the functional stability of Melapt is uncertain because the 3D structure adopted by the Melapt aptamer when bound to melanopsin is difficult to ascertain. In addition, it can be difficult for DNA aptamers to reach the retina, especially if injected into the eye bulb intravenously (Kwak and D'Amico, 1992; Hosoya and Tomi, 2005; Repetto et al., 2010; Patane et al., 2011); also, the stability of aptamers in blood and other body fluids will need to be assessed (Ma et al., 2021). Despite these limitations, Melapts could contribute to future research focused on resetting the phase of circadian clocks. Melapts could help us to better adapt to modern social life cycles, allow crops and domestic animals to be improved for greater productivity, and help shift workers in overcoming social jet lag by adjusting the phases of the circadian clock (Davidson et al., 2006; Saksvik et al., 2011; Barclay et al., 2012; Vetter et al., 2015; Li et al., 2021). These Melapts could contribute to resetting the phase of circadian clocks in photic input pathways.

## Data availability statement

The datasets presented in this study can be found in online repositories. The names of the repository/repositories and accession number(s) can be found at: https://www.ncbi.nlm.nih.gov/genbank/, AF147789.1.

## Ethics statement

The animal studies were approved by Animal care and use committee of Toyohashi Tech University. The studies were conducted in accordance with the local legislation and institutional requirements. Written informed consent was obtained from the owners for the participation of their animals in this study.

## Author contributions

RN and KN conducted the experiments, analyzed the data, performed the statistical analyses, prepared the figures, and contributed to the manuscript writing. MM assisted the experiments. YN established the photo-responsible cell line, while YK and YN conducted experiments with these cells. We are grateful to YK and YN for their supervision and helpful discussion. All authors contributed to the article and approved the submitted version.
