# Supplementary material for: Melanopsin DNA aptamers can regulate input signals of mammalian circadian rhythms by altering the phase of the molecular clock
Source: Front Neurosci. 2024 Apr 17;18:1186677. doi: 10.3389/fnins.2024.1186677 (PMC11062245; doi:10.3389/fnins.2024.1186677)
Supplement: Supplementary file 1 [file Data_Sheet_1.pdf]

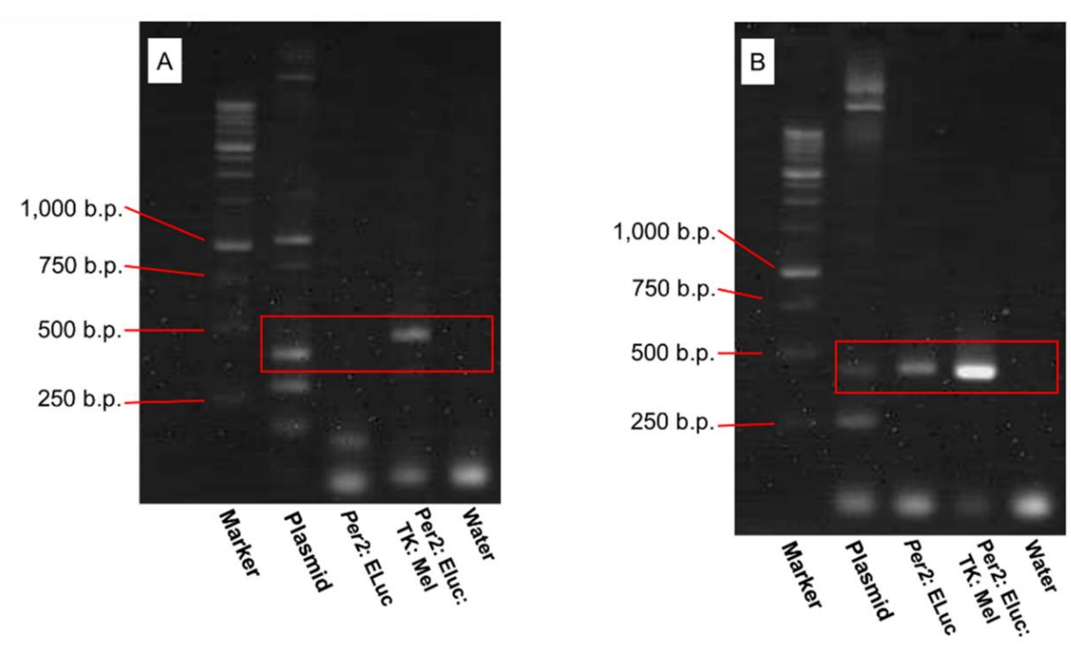

Figure S1 Expression of Melanopsin in *Per2:ELuc:*TK:Mel Cells confirmed by RT-PCR.

From left: markers, introduced Melanopsin TK plasmid (positive control), *Per2:ELuc* cells, *Per2:ELuc:*TK:Mel stable cells, water (negative control). (A) Electrophoresis results confirming Melanopsin expression. (B) Electrophoresis results confirming Glyceraldehyde-3-phosphate dehydrogenase (GAPDH) expression.

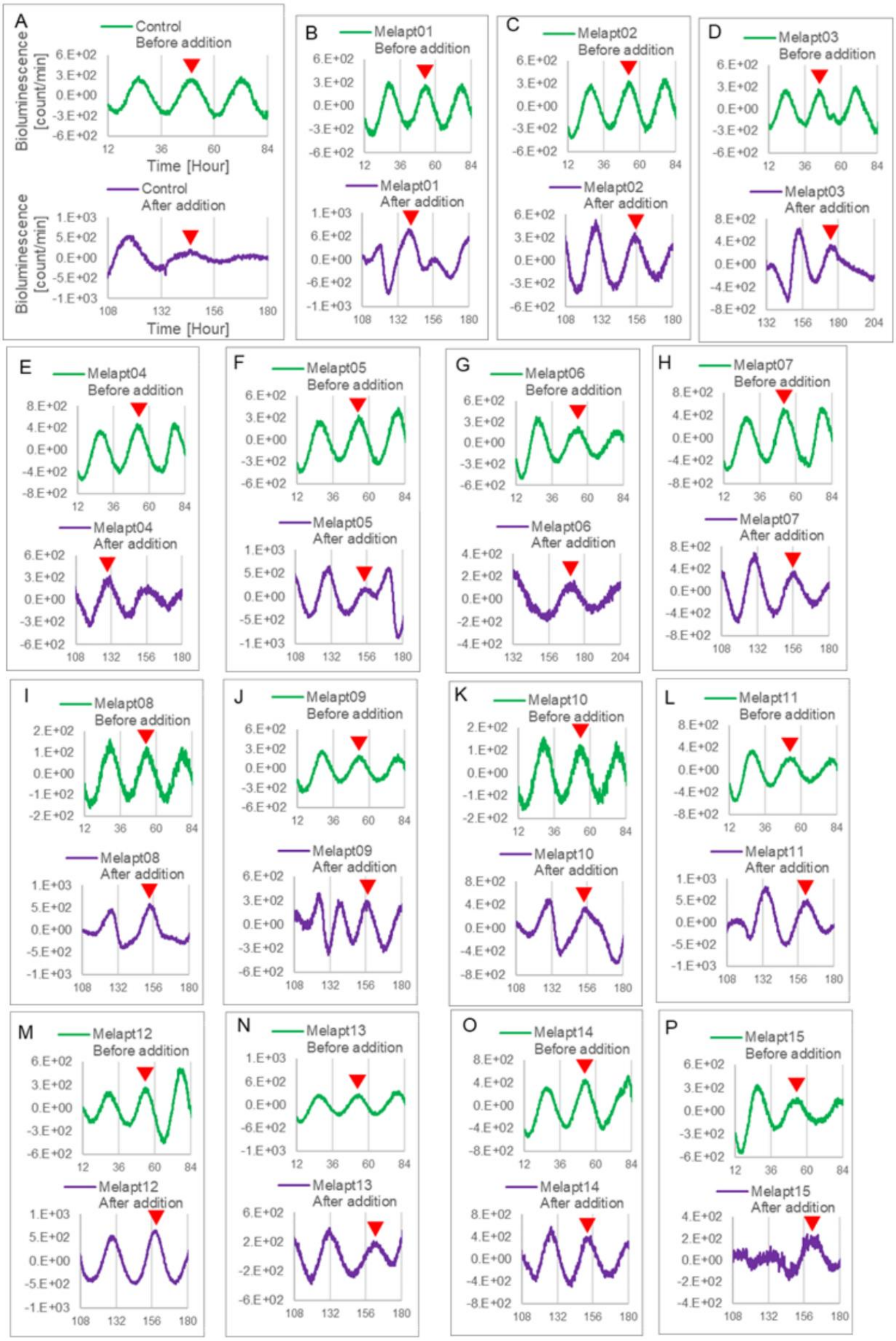
A
Control Before addition
Control After addition
Bioluminescence [count/min]
Time [Hour]
B
Melapt01 Before addition
Melapt01 After addition
C
Melapt02 Before addition
Melapt02 After addition
D
Melapt03 Before addition
Melapt03 After addition
E
Melapt04 Before addition
Melapt04 After addition
F
Melapt05 Before addition
Melapt05 After addition
G
Melapt06 Before addition
Melapt06 After addition
H
Melapt07 Before addition
Melapt07 After addition
I
Melapt08 Before addition
Melapt08 After addition
J
Melapt09 Before addition
Melapt09 After addition
K
Melapt10 Before addition
Melapt10 After addition
L
Melapt11 Before addition
Melapt11 After addition
M
Melapt12 Before addition
Melapt12 After addition
N
Melapt13 Before addition
Melapt13 After addition
O
Melapt14 Before addition
Melapt14 After addition
P
Melapt15 Before addition
Melapt15 After addition

Figure S2 Changeable *Per2:ELuc* emission rhythms before and after adding each Melapt at CT22.

(A) *Per2:ELuc* emission upon adding PBS (negative control). (B−P) *Per2:ELuc* emission upon adding Melapts 01–1. Upper row (green), *Per2:ELuc* emission before addition of Melapts (amplitude = 3). Lower row (purple), *Per2:ELuc* emission after adding Melapts observed for 3 days. Red triangles mark the peak.

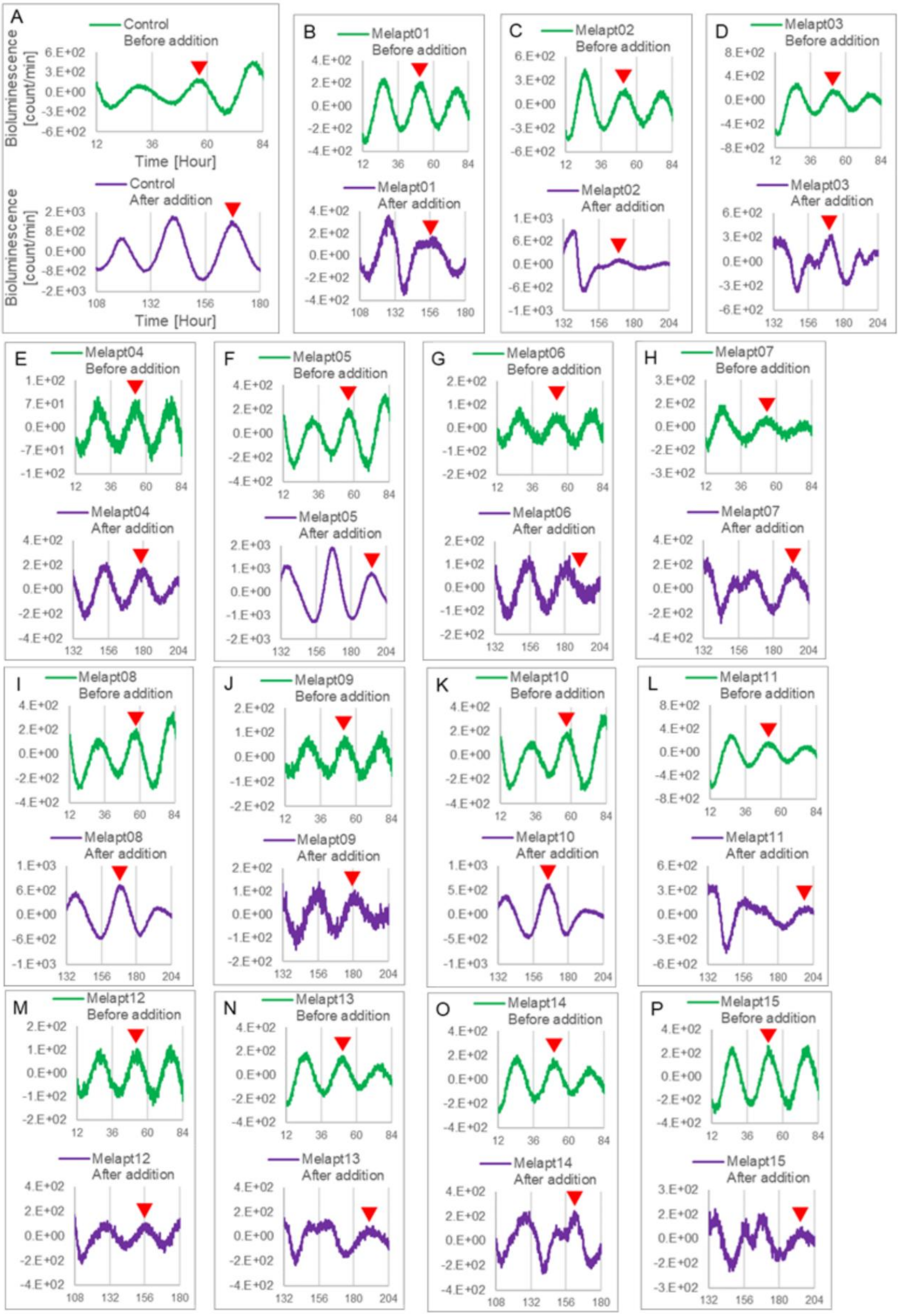
A
Control
Before addition
Bioluminescence [count/min]
Time [Hour]
Control
After addition
B
Melapt01
Before addition
Melapt01
After addition
C
Melapt02
Before addition
Melapt02
After addition
D
Melapt03
Before addition
Melapt03
After addition
E
Melapt04
Before addition
Melapt04
After addition
F
Melapt05
Before addition
Melapt05
After addition
G
Melapt06
Before addition
Melapt06
After addition
H
Melapt07
Before addition
Melapt07
After addition
I
Melapt08
Before addition
Melapt08
After addition
J
Melapt09
Before addition
Melapt09
After addition
K
Melapt10
Before addition
Melapt10
After addition
L
Melapt11
Before addition
Melapt11
After addition
M
Melapt12
Before addition
Melapt12
After addition
N
Melapt13
Before addition
Melapt13
After addition
O
Melapt14
Before addition
Melapt14
After addition
P
Melapt15
Before addition
Melapt15
After addition

Figure S3 Changes in *Per2:ELuc* emission rhythms before and after adding Melapts at CT8.

(A) *Per2:ELuc* emission upon adding PBS (negative control). (B–P) *Per2:ELuc* emission upon adding Melapts 01–1. Upper row (green), *Per2:ELuc* emission before addition of Melapts (amplitude = 3). Lower row (purple), *Per2:ELuc* emission after addition of Melapts observed for 3 days. Red triangles mark the peak.

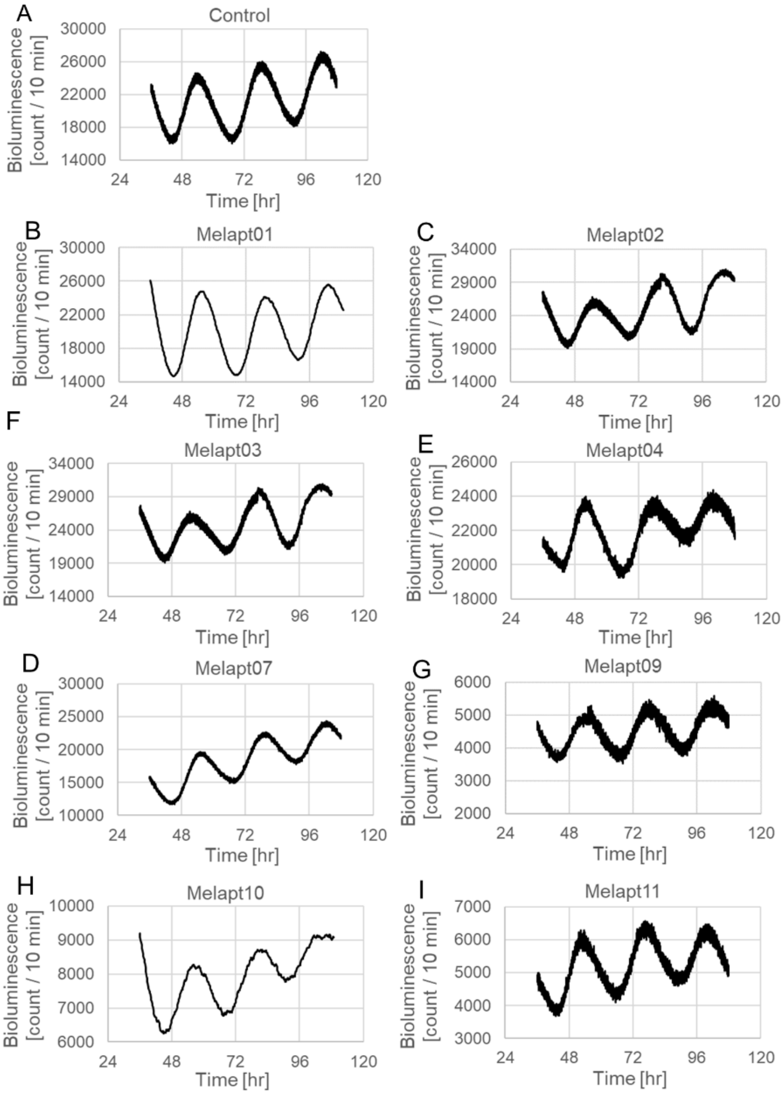

Figure S4 Phase shifts measured from *Per1::luc* expression rhythms in SCN slices from Melapt-injected *Per1::luc* Tg mice.

(A) Controls. (B) Injected with Melapt 01. (C) Melapt 02. (D) Melapt 03. (E) Melapt 04. (F) Melapt 07. (G) Melapt 09. (H) Melapt 10. (I) Melapt 11. Amplitude was measured at 36–108 h.
